# Exploring Psychotherapists’ Attitudes on Internet- and Mobile-Based Interventions in Germany: Thematic Analysis

**DOI:** 10.2196/51832

**Published:** 2024-11-07

**Authors:** Anne Sophie Hildebrand, Jari Planert, Alla Machulska, Lena Maria Margraf, Kati Roesmann, Tim Klucken

**Affiliations:** 1 Department of Clinical Psychology and Psychotherapy University of Siegen Siegen Germany; 2 Unit of Clinical Psychology and Psychotherapy in Childhood and Adolescence Department of Psychology University of Osnabrück Osnabrück Germany

**Keywords:** eHealth, psychotherapy, psychotherapists’ perspectives, thematic analysis, internet- and mobile-based intervention

## Abstract

**Background:**

In recent years, internet- and mobile-based interventions (IMIs) have become increasingly relevant in mental health care and have sparked societal debates. Psychotherapists’ perspectives are essential for identifying potential opportunities for improvement, facilitating conditions, and barriers to the implementation of these interventions.

**Objective:**

This study aims to explore psychotherapists’ perspectives on opportunities for improvement, facilitating conditions, and barriers to using IMIs.

**Methods:**

The study used a qualitative research design, utilizing open-ended items in a cross-sectional survey. A total of 350 psychotherapists were asked to provide their written opinions on various aspects of IMIs. Thematic analysis was conducted to analyze the data and identify core themes.

**Results:**

The analysis revealed 11 core themes related to the use of IMIs, which were categorized into 4 superordinate categories: “Applicability,” “Treatment Resources,” “Technology,” and “Perceived Risks and Barriers.” While many psychotherapists viewed IMIs as a valuable support for conventional psychotherapy, they expressed skepticism about using IMIs as a substitute. Several factors were perceived as hindrances to the applicability of IMIs in clinical practice, including technological issues, subjective concerns about potential data protection risks, a lack of individualization due to the manualized nature of most IMIs, and the high time and financial costs for both psychotherapists and patients. They expressed a desire for easily accessible information on evidence and programs to reduce the time and effort required for training and advocated for this information to be integrated into the conceptualization of new IMIs.

**Conclusions:**

The findings of this study emphasize the importance of considering psychotherapists’ attitudes in the development, evaluation, and implementation of IMIs. This study revealed that psychotherapists recognized both the opportunities and risks associated with the use of IMIs, with most agreeing that IMIs serve as a tool to support traditional psychotherapy rather than as a substitute for it. Furthermore, it is essential to involve psychotherapists in discussions about IMIs specifically, as well as in the development of new methodologies in psychotherapy more broadly. Overall, this study can advance the use of IMIs in mental health care and contribute to the ongoing societal debate surrounding these interventions.

## Introduction

In recent years, eHealth has gained significant importance in the field of psychotherapy. Part of this trend is the development of internet- and mobile-based interventions (IMIs) targeting various somatic diseases and psychological disorders [1]. These interventions consist of self-guided apps and different levels of psychotherapeutic support (eg, stand-alone or blended care approaches [1-3]) that are readily accessible almost anywhere and anytime [4]. In Germany, health insurance covers the costs of certain IMIs in the form of mobile apps (Digitale Gesundheitsanwendungen) after they have been approved by the Federal Institute for Drugs and Medical Devices [5]. Technological advancements such as virtual reality, gamification techniques, wearable devices, and chatbots can further expand the applicability of IMIs and the range of users who can benefit from them [3,6-8].

Although the effectiveness and efficacy of some IMIs require further examination, others have been shown to reduce symptom severity [9-11]. While many IMIs incorporate basic cognitive behavioral therapy (CBT) interventions [2,3,8], studies have found psychodynamic approaches to be promising [12,13].

The main goal behind the development of IMIs is to overcome barriers to mental health care access (eg, the global treatment gap [14]). This became particularly important during the COVID-19 pandemic, when treatments that did not require in-person contact, such as video therapy and IMIs, were essential to prevent the spread of infections [15-17]. Previous studies have identified several challenges associated with video therapy, including establishing emotional connections, managing distractions during sessions, ensuring patient privacy, and setting boundaries for therapists [15]. Depending on the country’s insurance system, the financial aspect affects not only the individual but also has a broader societal impact. IMIs have the potential to improve the cost-effectiveness of care, optimize resource distribution, and reduce the burden on the mental health care system [18,19]. During the COVID-19 pandemic, digital treatment options were particularly viewed as a means of providing accessible and safe mental health care. At the same time, it is important to emphasize that, despite the promising opportunities and existing facilitating conditions, there may also be potential risks associated with the implementation and use of IMIs. Concerns that have been previously identified include a lack of direct professional involvement and the accompanying risk of misuse or harm, particularly in cases of severe or comorbid mental health conditions [20,21]. As the use of IMIs increases, potentially altering the provision of mental health care, their future will be shaped by clinical research on both treatment efficacy and the experiences of all stakeholders.

While there is a substantial body of research on the efficacy of IMIs [21] and how patients can benefit from their use [22], comparatively less is known about how psychotherapists perceive the use of IMIs in their professional practice. This gap is concerning, given that the rise of IMIs has significantly impacted psychotherapists’ work. Previous research on psychotherapists’ perspectives on certain IMIs suggests that they may view these tools as a welcomed support [23]. As such, IMIs have been identified as tools that could help psychotherapists gain more control over their work time [24] or be integrated into certain psychotherapeutic interventions [25,26]. However, concerns have also been raised about the potential role of IMIs in therapy [23]. For instance, some worry that IMIs could be seen as substitutes for professional treatment or that they may reduce the personalized therapist-patient alliance to a more mechanistic, algorithm-driven interaction [20,23]. While these studies provided valuable insights, most of the implications were derived from trials focusing on specific IMIs, raising doubts about whether the findings can be generalized to a broader population of psychotherapists who encounter a wide variety of IMIs in their daily practice. For the successful integration of technological aids into psychotherapy, it is essential to incorporate the opinions and experiences of psychotherapists, as the use of IMIs brings up numerous professional and ethical considerations.

Existing studies investigate how psychotherapists perceive the use of IMIs [20,23]. However, these studies often have small sample sizes or are limited to specific interventions in randomized controlled trials [20]. As IMIs are still viewed with uncertainty by psychotherapists, their opinions require further comprehensive investigation. This necessitates larger samples and a diversity of psychotherapeutic orientations and scenarios in the application of IMIs. This will provide a robust accumulation of attitudes, ultimately identifying the challenges and risks associated with IMIs and facilitating their further development and integration into psychotherapeutic practice. This study aims to explore the attitudes and opinions of various psychotherapists regarding IMIs in a bottom-up fashion. As psychotherapists are among the primary providers of IMIs, the results of this study can help ensure that their considerations are represented in the design of future IMIs. This can potentially facilitate the use and applicability of the respective interventions. To achieve this goal, psychotherapists were invited to complete an online survey featuring open-ended questions about their attitudes toward various aspects of IMIs. Their responses were subsequently analyzed using thematic analysis.

## Methods

### Participants and Procedure

For recruitment, licensed psychotherapists and psychotherapists in training from any approved psychotherapeutic specialization supported by German health insurance (such as CBT, psychodynamic therapy, analytical psychotherapy, and systemic psychotherapy) were contacted via email. This outreach utilized distribution lists from universities, training institutes, and professional associations, as well as colleague networks and social media. This contact included information about the study design, research topic, and a link to access the online survey. Upon opening the survey link, participants were provided with detailed information, and written informed consent was obtained. A total of 350 psychotherapists anonymously completed the questionnaire using Limesurvey 3.28.18 (Limesurvey GmbH). After completing the survey, participants had the opportunity to enter a draw for 3 spots in a workshop on digitization and technical security in psychotherapy at the University of Siegen. No additional compensation was provided.

### Materials

The survey included demographic questions to assess participants’ age, gender, license, psychotherapeutic orientation, and workplace setting (eg, inpatient and outpatient settings), as well as questions regarding prior usage of IMIs (for questions on prior usage, see Table S1 in Multimedia Appendix 1). To explore psychotherapists’ attitudes toward IMIs, 14 open-ended questions were administered (for a selection of the administered questions, see Textbox 1; for the full list, see Table S1 in Multimedia Appendix 1). These questions covered topics such as social influence, barriers, risks and challenges, facilitating conditions, opportunities, desirable functions, and concerns related to psychotherapeutic orientations. The questions were based on the Unified Theory of Acceptance and Use of Technology [27,28], which incorporates components such as *social influence* and *facilitating conditions*. We also addressed negative aspects of *performance expectancy* and *effort expectancy* by asking about challenges, requirements, and deficiencies. This approach allowed us to gather findings without overly restricting the investigation. All items were presented in German and reviewed by 2 independent clinical psychologists for face validity (see Figure S1 in Multimedia Appendix 1).

**Examples of the open questions concerning psychotherapists’ attitudes toward internet- and mobile-based interventions, administered in an online survey between 2021 and 2022.**

- Does your working environment support the use of new technologies and if so, how?
- Was there something that kept you from using internet- and mobile-based interventions (IMIs) in your psychotherapeutic practice and if so, what?
- What challenges could the use of IMIs encounter?
- Which requirements need to be met for you to use IMIs in your psychotherapeutic practice?
- What functions should IMIs include to become useful for you in psychotherapy?
- Are there psychotherapeutic orientations for which IMIs are (particularly/rather not) suited and if so, which?

Note: The textbox presents a selection of the administered questions. All questions and a definition of internet- and mobile-based interventions given to psychotherapists can be found in Table S1 in Multimedia Appendix 1.

### Ethical Considerations

The study protocol (12/2021) was approved by the Ethics Committee of the University of Siegen (reference number LS_ER_57). Data collection occurred in 2021 and 2022 during the COVID-19 pandemic. Informed consent was obtained from each participant before enrollment. Participation was entirely voluntary, and participants had the right to withdraw their consent at any time. Data were anonymized using a trial identification number. The data were saved on a secure, self-encrypting database and were accessible only to the responsible researchers. As a result of pseudonymization, individual participants could not be identified, and no personal information or images were collected. Participants were not financially compensated for their participation; however, they had the opportunity to enter a draw for 3 spots in a workshop on digitization and technical security in psychotherapy at the University of Siegen.

### Data Analysis

For the demographic variables, means, SDs, and frequencies were calculated. A thematic analysis was conducted using the responses of psychotherapists to the open survey items [25,26]. All answers were sorted according to the participants’ responses and entered into the text analysis program MAXQDA (VERBI). Two independent coders (ASH and JP) followed a step-by-step analysis procedure for psychological thematic analysis [29,30]. The first coder reviewed all entries and created different codes for the answer categories from the data in an inductive manner. The second coder then assigned any remaining data entries to these codes. Codes were structured based on their content rather than the questions from which they were derived. This meant that 2 data entries could be assigned the same code, even if they originated from different questions, as long as their content matched. The second coder merged codes into higher-ranked themes (referred to later as core themes) where applicable. Afterward, both coders diligently reviewed the coding system, its categories, and the corresponding entries. As the analysis method used is exploratory, no research hypotheses or predictions were established beforehand. Both coders defined and interpreted the core themes and subthemes along with their corresponding codes. The quotations used in the results sections have been translated from German to English and checked for accuracy.

## Results

### Demographics

The sample (N=350) consisted of 80 (22.9%) male and 267 (76.3%) female participants, with a mean age of 42.8 (SD 12.2) years. The median completion time for the survey was 17 minutes. For more information, see Table 1.

**Table 1:** Demographic description of the study sample in this qualitative study conducted between 2021 and 2022 in Germany (N=350 psychotherapists).

Demographics			Total		Female		Male
Participants^a^, n (%)			350 (100.0)		267 (76.3)		80 (22.9)
Age, mean (SD)			42.8 (12.2)		41.8 (11.8)		46.3 (13.3)
Licensed, n (%)			269 (76.9)		203 (76.0)		63 (78.8)
Therapeutic orientation, n (%)							
	Cognitive behavioral therapy	303 (86.6)		232 (86.9)		68 (85.0)	
	Psychodynamic therapy	30 (8.6)		21 (7.9)		9 (11.3)	
	Analytical psychotherapy	9 (2.6)		7 (2.6)		2 (2.5)	
	Systemic	3 (0.9)		3 (1.1)		0 (0)	
	Other	5 (1.4)		4 (1.5)		1 (1.3)	
Therapeutic setting, n (%)							
	Outpatient	304 (86.9)		236 (88.4)		69 (86.3)	
	Inpatient	16 (4.6)		9 (3.4)		5 (6.3)	
	Other	6 (1.7)		4 (1.5)		0	
	Multiple	24 (6.9)		18 (6.7)		6 (7.5)	

^a^Of the 350 participants, 3 did not report their gender.

Compared with data from the Federal Health Monitoring System in Germany, the sample of licensed psychotherapists in this study was representative in terms of gender (female: 267/350, 76.3%; male: 80/350, 22.9%; and 3/350, 0.8% who did not answer the question at all) and work setting (outpatient: 304/350, 86.9%, with 236/267, 88.4%, females and 69/80, 86.3%, males) of licensed German psychotherapists [31].

### Prior Experience With IMIs

Regarding the use of IMIs, 92 of the 350 (26.3%) psychotherapists reported already prescribing them, while 221 (63.1%) had not yet used IMIs. Overall, these psychotherapists reported 772 prescriptions (see Multimedia Appendix 2 for details regarding the different prescriptions). They also estimated a dropout rate of 48.39% for IMIs, which was slightly higher than the dropout rates of approximately 40% observed in conventional psychotherapy studies [32,33].

### Thematic Analysis

#### Overview

**Figure 1:**
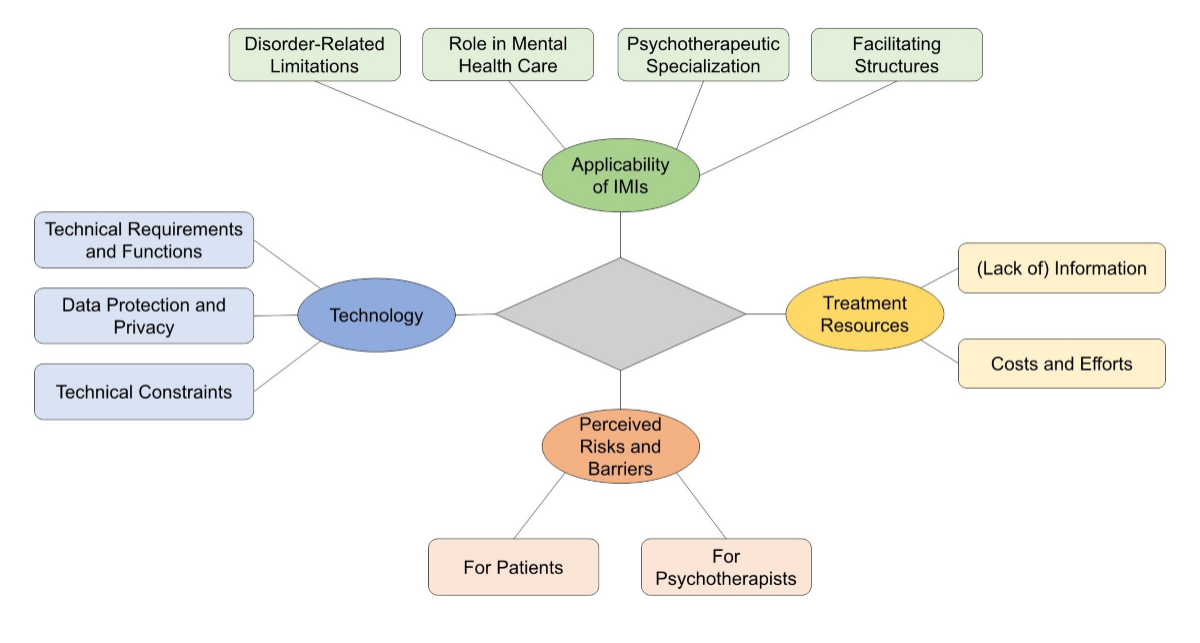
A thematic map of the 4 categories and the respective core themes. IMI: internet- and mobile-based intervention.

Through the thematic analysis of responses to open questions from 350 psychotherapists in this survey, 11 core themes emerged: (1) Disorder-Related Limitations, (2) Facilitating Structures, (3) Psychotherapeutic Specialization, (4) Role in Mental Health Care, (5) Lack of Information, (6) Costs and Efforts, (7) Technical Constraints, (8) Technical Requirements and Functions, (9) Data Protection and Privacy, (10) Perceived Risks and Barriers for Patients, and (11) Perceived Risks and Barriers for Psychotherapists. These themes were sorted into 4 overarching categories: Applicability of IMIs, Treatment Resources, Technology, and Perceived Risks and Barriers. Overall, psychotherapists expressed mixed opinions and experiences regarding the use of IMIs. Quotations from the responses provided by psychotherapists to the survey items appear in italics in the respective sections. Figure 1 offers detailed information regarding the 4 identified categories and their respective core themes (see also Tables S2-S4 in Multimedia Appendix 1).

#### Applicability of IMIs

##### Disorder-Related Limitations

Psychotherapists have expressed concerns about the limited applicability of disorder-related treatments. They felt that IMIs were least suitable for treating psychosis and personality disorders.

Personality disorders, for example. I fear that the relational component is missing in order to actually make a change. Apart from that, for me, it depends on the severity of the disorder. As an additional tool during psychotherapy, I would not exclude any group of disorders.

T139, female, age 29 years

Several psychotherapists also mentioned (severe) depression, posttraumatic stress disorder, relationship disorders (eg, issues with social interactions or intimate sexual contact), anxiety disorders, dissociative disorders, addiction, and bipolar disorder.

Psychosis, personality disorders, many forms of depression with a biographical-traumatic origin, and obsessive-compulsive disorders - all require direct human contact for effective treatment.

T217, male, age 23 years

As general characteristics, psychotherapists mentioned suicidality, acute crises, comorbidity, high complexity and severity, and a lack of cognitive abilities as circumstances under which they would refrain from using IMIs in their therapy. For certain disorders (eg, social anxiety disorder, obsessive-compulsive disorder), they feared that IMIs might even reinforce avoidance behavior.

*[Using IMIs]could be part of an avoidance strategy. and are basically [not suited] for all disorders, where the bond with the therapist is central*.

T129, female, age 32 years

By contrast, psychotherapists indicated that IMIs could be especially suitable for patients with mild symptom severity or for those who are highly motivated and autonomous. Overall, most psychotherapists reported that suitability should be determined on a case-by-case basis and not rely solely on the diagnosed disorder(s).

##### Facilitating Structures

Therapists also commented on the structures that facilitate the applicability of IMIs. Most participating psychotherapists stated that their employers already supported the use of new technology in therapy. This support included enabling video therapy, offering technical assistance and training programs, and providing the necessary software and hardware. Some employers even implemented feedback systems, online diagnostics, and promoted digitalization in psychotherapy. Additionally, health insurance companies were viewed as supportive of computer-based therapy, as they compensated patients for costs related to video therapy and IMIs. As 304 of the 350 (86.9%) psychotherapists worked in independent practices, they had the autonomy to decide whether to integrate IMIs and video therapy into their therapeutic work.

I am self-employed and open to supportive and data-protected technologies.

T37, female, age 56 years

This situation not only provides them with opportunities to use IMIs but also requires them to take responsibility for their implementation. Particularly for self-employed psychotherapists, the lack of financial compensation for time spent on training in the respective IMIs was a significant issue.

Getting accustomed to the apps costs time and money.

T273, female, age 41 years

While several psychotherapists expressed openness to using IMIs in their practices and discussing them with colleagues, others explicitly stated their lack of support. They cited issues related to compensation, personal preference, or ideological reasons.

##### Psychotherapeutic Specialization

Most psychotherapists felt that IMIs would be most applicable in CBT interventions compared with other forms of therapy. Psychoanalysis was considered the least suitable for IMIs, closely followed by psychodynamic therapy. This perception was attributed to the specific techniques used in these approaches and the emphasis on the therapeutic relationship in treating disorders.

[IMIs] are better suited for CBT than for psychodynamic or analytic therapy, as for the latter, the personal relationship is more important than in CBT.

T159, female, age 30 years

Together with the patient, the therapist can acquire and realize unconscious conflicts better than an app.

T38, female, age 35 years

Systemic therapy was also viewed as less appropriate for IMIs, given the importance of relationship systems in psychotherapeutic interventions.

##### Role in Mental Health Care

Many psychotherapists addressed the integration of IMIs in mental health care and their role within it. They viewed IMIs as a valuable addition to conducting therapeutic exercises at home or as a tool for bridging waiting times, such as by providing first-line psychoeducation and an introduction to therapy.

[IMIs] probably have the potential to help between the intake session and the start of the treatment in order to prepare the therapy. Sadly, they are misused as a political tool in order to disguise the shortage of treatment capacities. Simply because of that, it is, in my opinion, damaging both for patients and therapists to use [IMIs], as rather nothing will change about the shortage of treatment capacities.

T325, male, age 34 years

[IMIs] are a great supplement to psychotherapy.

T54, female, age 34 years

However, it has been criticized that governmental institutions do not clearly communicate the goals that should guide the development of IMIs. The majority of psychotherapists cannot envision IMIs as an effective substitute for face-to-face psychotherapy and believe they should not be perceived as such.

As a stand-alone, it is rather a low-threshold service for prevention.

T54, female, age 34 years

In fact, psychotherapists expressed concern that IMIs might be intended to further reduce mental health expenses for insurance companies by substituting therapy sessions.

There is too much focus on quickly treating as many people as possible instead of considering the cause of the high increase of psychological disorders. For example, precarious work conditions, isolation, or anonymity.

T20, female, age 33 years

It was emphasized that IMIs could not replace the therapeutic relationship, which “is known to be an important effect factor for the success of psychotherapy” [T87, female, age 36 years]. Psychotherapists felt that IMIs—especially when used as stand-alone treatments—might offer a more economically driven solution to the rising demand for psychotherapy. However, this could result in a reduction of psychotherapists funded by health insurance and a decrease in their perceived value.

#### Treatment Resources

##### Lack of Information

One core theme that emerged was the “Lack of Information” regarding IMIs. Psychotherapists reported a deficiency in knowledge and available information on 2 main aspects: (1) the content and applicability of IMIs, and (2) the efficacy of IMIs. Acquiring knowledge about the content and applicability was perceived as requiring significant effort, as this information is often only available through specific seminars or by contacting developers directly.

Unless a provider is contacted directly, only an introduction or information material is handed out, and, if necessary, a trial access is granted. There is little detailed information online about the IMIs, so that patients cannot really be well informed.

T299, female, age 37 years

The lack of information was particularly problematic, especially considering the perceived number of available apps. Furthermore, psychotherapists noted that the scientific foundation of IMIs was not well communicated.

[There is a] lack of or insufficient evidence of effectivenes.

T91, female, age 41 years

This lack of information hindered a feasible transfer into psychotherapeutic practice. As a possible solution, many participants suggested the need for more readily accessible information, such as short presentation videos, tutorials, booklets, and recommendation systems. Others emphasized the importance of increased training opportunities and access to apps for testing. Additionally, some expressed concern about false information and misleading advertisements, which could elevate patients’ expectations and ultimately lead to disappointment.

##### Costs and Efforts

Psychotherapists identified high costs and efforts as another core theme. They expressed that a significant amount of time and resources is required for initial training, prescription, and accounting.

It is an additional effort in an already loaded workday.

T155, male, age 57 years

This was perceived as problematic because health insurance companies do not compensate psychotherapists for the hours spent on these tasks. Psychotherapists expressed concerns that economic interests might take precedence over patients’ needs.

Economic interests/lobbyism are so high that scientific evaluation and user experiences are annulled. Regarding the limited resources in the health care system, this is not justifiable.

T272, male, age 40 years

Psychotherapists expressed a desire for more support from insurance companies, including compensation for the time they invest in testing, prioritizing patients’ needs, making apps more affordable, and simplifying the prescription processes. In addition to needing more accessible information, psychotherapists emphasized the necessity for “permanent, free, and complete access for psychotherapists” [T228, women, age 31 years] to adequately assess the respective IMIs.

#### Technology

##### Technical Constraints

Another core theme that emerged was “Technical Constraints” that interfere with the treatment experience. Psychotherapists expressed concerns about the unequal digital infrastructure among patients, which could complicate the effective use of IMIs.

Part of my patients (countryside, elderly) do not own a smartphone or are not really familiar with it. The quality of the internet connection still is lacking on the country-side. Unbelievable, in 2022.

T200, male, age 65 years

Additionally, it was noted that the use of IMIs might result in increased screen time.

Many of my patients spend a lot of time with technical devices. Oftentimes, the private smartphone is used for occupational matters, so that they never [relax]. If now a treatment app additionally is used digitally, it again is a reach to the smartphone and a missing break for the brain.

T114, female, age 43 years

Psychotherapists emphasized that technical issues would take up time that could otherwise be dedicated to direct patient contact.

Time and content is going missing when technical problems arise and it takes space that deflects the attention away [from therapy].

T277, female, age 34 years

##### Technical Requirements and Functions

Psychotherapists expressed that IMIs should incorporate specific features as technical requirements and functions. They emphasized 3 crucial aspects: (1) high usability, (2) customization options, and (3) data protection.

First, they deemed it essential for the user interface to be “as intuitive as possible” [T90, female, age 54 years] to enhance the applicability of IMIs.

Second, they emphasized the necessity for IMIs to be customizable. Psychotherapists expressed the desire for the components of IMIs to be tailored to each patient’s individual needs, such as through a modular system that allows psychotherapists and patients to select specific modules and exercises.

A possibility is required to assemble an individual program (relaxation, mindfulness, etc.).

T140, female, age 29 years

They also requested customization options for the user interface, including different voice options for tutorials and modules, as well as support for multiple languages.

Third, psychotherapists expressed concerns about data protection. They emphasized the need to ensure that patients’ data are kept safe and secure, with measures in place to prevent misuse. Additionally, they highlighted the importance of providing an option for patients to fully delete their data if needed. Psychotherapists indicated that patients should have complete control over their data and that informed consent must be obtained before using any patient data.

Patients should have the option to give access to their therapists regarding their learning status or symptom diary.

T32, female, age 37 years

Some psychotherapists also suggested that IMIs should incorporate components that facilitate interaction between psychotherapists and patients, such as diaries and protocols, video or audio recordings, feedback systems, and emergency functions (see Table S4 in Multimedia Appendix 1). Regarding the platform, some psychotherapists preferred web-based apps that are accessible from both PCs and smartphones, while others favored apps specifically designed for smartphones or tablets.

##### Data Protection and Privacy

Psychotherapists expressed concerns regarding data protection, specifically worrying about the adequacy of security measures in treatment apps. They noted that most IMIs are exclusively available on the distribution platforms of the 2 largest mobile phone operating systems.

Data stored in a system with internet access could always be stolen or misused.

T279, female, age 38 years

Psychotherapists noted that it is often unclear who is authorized to collect patient data and for what purposes, as these regulations are set by the distribution platform provider rather than the developer of the treatment app. Consequently, third parties could potentially gain access to sensitive patient data, such as information from a user who downloads an app designed to reduce alcohol consumption.

The data protection guidelines for outpatient centers are reasonably high as it has to be made sure that the data do not land on [foreign] servers. Yet, I should encourage patients to download smartphone apps from [distribution platforms], without it being made sure that [companies] do not process the information that [patient X] has panic or that [patient Y] drinks too much? From now on, I will only make use of web-based apps.

T204, male, age 53 years

Consequently, many psychotherapists have expressed a desire for robust data protection measures and greater transparency.

#### Perceived Risks and Barriers

##### Perceived Risks and Barriers for Psychotherapists

Psychotherapists have reported that the integration of IMIs has affected their work with patients. Specifically, several psychotherapists noted a decreased sense of control and self-efficacy in their practice due to the incorporation of IMIs.

Psychotherapists are not involved in the process, I could be held responsible if something goes wrong in regard to treatment errors.

T37, female, age 56 years

Many psychotherapists mentioned that the primary reason for not integrating IMIs into their daily practice was the reduced contact between patients and therapists, which they perceived as impersonal. Two potential solutions were proposed: the first was to involve psychotherapists in the therapeutic process, such as through blended therapy; the second was to include therapists in the design and conceptualization of IMIs. Additionally, concerns were frequently raised about unclear legal responsibilities when prescribing IMIs.

I would be afraid that patients sue me if something does not work out the way it should.

T168, female, age 37 years

Psychotherapists expressed concerns about liability in emergencies and cases of patient deterioration, such as acute crises or suicidality. Often, uncertainties regarding potential claims for recourse contributed to their hesitance to utilize IMIs.

##### Perceived Risks and Barriers for Patients

Psychotherapists also highlighted barriers, such as technical requirements, age, vision impairments, and somatic disorders, that could hinder patients from effectively using IMIs. In particular, they expressed concerns about the highly heterogeneous patient population and the lack of individualization and suitability of apps for specific patients.

Many older patients do not have the technical access.

T164, female, age 28 years

Another factor noted was the absence of therapeutic relationships in computerized treatment. Most psychotherapists argued that this could lead to poor compliance and foster feelings of rejection, ultimately resulting in patients withdrawing from therapy.

The use of the apps among my clients has fizzled out, as the treatment motivation has drastically decreased after a few weeks.

T201, female, age 58 years

Moreover, particularly when used as stand-alone treatments, IMIs may provide unsuitable or misleading information and could be utilized inappropriately.

Initiating suicidality or endangerment of others could be detected too late.

T23, female, age 40 years

Psychotherapists express concern about a “higher responsibility for the patient and the increasing isolation and deindividualization” [T90, female, age 54 years]. In the context of blended therapy, the accompanying apps may overwhelm patients, as they would need to complete additional modules and spend more time practicing at home.

## Discussion

### Principal Findings

This study aimed to explore psychotherapists’ attitudes toward IMIs. As psychotherapists are among the primary providers of IMIs, it is essential to incorporate their opinions and experiences. This incorporation will further facilitate the successful integration of such technological aids in psychotherapy. The thematic analysis generated 11 core themes, which were organized into 4 overarching categories: Category 1 is the Applicability of IMIs. It covers themes such as Disorder-Related Limitations, Facilitating Structures, Psychotherapeutic Specialization, and Role in Mental Health care. Category 2 is Treatment Resources. It has 2 themes, which are Lack of Information and Costs and Efforts. Category 3 is Technology. It covers themes such as Technical Constraints, Technical Requirements and Functions, and Data Protection and Privacy. Category 4 is Perceived Risks and Barriers. It covers the themes of Perceived Risks and Barriers (1) for Psychotherapists and (2) for Patients. The opinions gathered across the different core themes highlighted current shortcomings and barriers that hinder the use of IMIs, as well as facilitating conditions and ideas for further development.

### Applicability of IMIs

The most prominent category was the psychotherapists’ ideas and concerns regarding the applicability of IMIs. In this category, perceived disorder-related apps and associated limitations were particularly notable. Psychotherapists agreed that IMIs could be integrated into clinical practices for certain therapies, especially for anxiety disorders and depression. Current meta-analyses and reviews support the effective implementation of IMIs in the treatment of anxiety disorders [21] and depression [22]. However, psychotherapists expressed concerns about using IMIs for the treatment of psychotic disorders, personality disorders, comorbid conditions, trauma-related issues, and severe disorders.

While this perception may be partly supported by current research highlighting the challenges of using IMIs for certain disorders, some findings suggest that IMIs can effectively treat severe comorbidities [34]. However, these findings also underscore the limitations of IMI-related research for mental disorders beyond anxiety and depressive disorders [34]. To reduce the uncertainty among psychotherapists, more studies are needed to test IMIs for treating these types of disorders or more complex populations. Another factor affecting the applicability of IMIs is the uncertainty surrounding their role in current and future mental health care, particularly whether they are intended to assist or substitute for in-person treatment. Many psychotherapists criticize the notion of replacing psychotherapy with treatment apps, which contributes to their reluctance to integrate IMIs into their practices. Therefore, political actors and leaders in the field of mental health care should engage in discussions to clarify the role of IMIs in psychotherapy. Another concern raised by psychotherapists that limits the applicability of IMIs is their therapeutic focus. While most psychotherapists considered IMIs suitable for CBT, they expressed doubts about the potential integration of these apps into other psychotherapeutic interventions. Previous research has highlighted several benefits of using IMIs across different psychotherapeutic orientations [35]. It can, therefore, be assumed that CBT-focused IMIs may also benefit patients undergoing psychodynamic or psychoanalytic treatment. Additionally, previous studies have confirmed symptom reduction when using IMIs within psychodynamic approaches [12,13]. For example, these approaches encouraged patients to reflect on emotional conflicts related to their symptoms [36]. Developing IMIs that integrate well into psychodynamic or psychoanalytic treatment approaches offers several benefits and economic advantages. However, it is important to note that contemporary psychotherapy is gradually shifting away from strict adherence to a single school of thought, incorporating a range of evidence-based practices (eg, see the Cognitive Behavioral Analysis System of Psychotherapy for chronic depression [37]). This positive development will also influence the design of future IMIs. Despite this, some psychotherapists have reported that measures facilitating the applicability of IMIs are already present in their work environments. This suggests that employers and insurance companies are supportive of IMIs in the mental health sector. However, psychotherapists are seeking additional support.

### Treatment Resources

Regarding the second most frequently addressed category, “Treatment Resources,” and its associated core themes, the results overwhelmingly suggest a perceived lack of information about IMIs. One potential reason is that psychotherapists may lack confidence in using IMIs, even if they were to try them. They noted that the anticipated costs and efforts outweigh the perceived benefits of incorporating IMIs into psychotherapy. To address this issue, psychotherapists suggested that they should be compensated for the initial training and the supervision of patients when using IMIs. They also recommended the creation of overarching platforms or reimbursed seminars where they could easily access the necessary information. Additionally, psychotherapists expressed concerns about the scientific foundation of IMIs in mental health care, noting that some IMIs are validated solely based on research conducted by the companies or their associated researchers [38]. Although studies have confirmed the benefits of IMIs for patients, such as reducing symptom severity [2,9-11], more research from independent institutions is needed to validate their use. Another concern raised by psychotherapists is the perceived lack of a solid scientific foundation, which may stem from the fact that study results are not consistently communicated to practicing clinicians. To bridge this potential science-practitioner gap, this study emphasizes the need for relevant information and training to be made more easily accessible.

### Technology

The technological aspects of IMIs, along with their associated core themes, formed the third category. This category highlights the technical shortcomings and past experiences psychotherapists have had with IMIs. Psychotherapists emphasized that the availability of high-quality IMIs is crucial for successfully integrating them into therapeutic practices, a finding consistent with previous qualitative studies on blended therapy in a German sample [20]. It was also important for psychotherapists to have the option to tailor the content of IMIs to meet the specific needs of individual patients, rather than using a one-size-fits-all approach, as highlighted by previous literature [25]. While psychotherapists had many ideas regarding the configuration of IMIs, they criticized that these concepts are not adequately represented in the apps.

Another critical concern was data protection and the need to ensure patient privacy. In Germany, where the survey was conducted, data protection guidelines for software developers of digital health apps are relatively strict. For instance, these apps cannot contain third-party advertisements [39]. However, downloading an IMI still exposes sensitive patient information to the distribution platform. This highlights the need for a centralized IMI distribution platform to prevent broader privacy issues from being regulated by third parties [39].

### Perceived Risks and Barriers

The fourth category of core themes, “Perceived Risks and Barriers,” included factors that the surveyed psychotherapists associated with potential risks or obstacles related to the use of IMIs, which could affect either themselves, their patients, or both. Many expressed concerns that the use of IMIs might make psychotherapy feel impersonal, potentially reducing its effectiveness, as interpersonal factors—such as the therapeutic relationship—are essential to the success of psychotherapy [40]. The results of our study also indicate that the barriers psychotherapists associate with the use of IMIs are similar to those identified in a systematic review focusing on barriers to conventional clinical practice [41]. In those studies, factors such as lack of knowledge, attitudes toward IMIs, and legal and resource-related issues were identified as limitations for professionals. To assess the impact of using IMIs, it is essential to include evaluations of adverse events and unwanted treatment reactions in future clinical trials investigating the efficacy of IMIs [42].

### Limitations

This study provides a comprehensive portrayal of the multifaceted attitudes of psychotherapists toward IMIs in mental health care. However, there are some limitations. First, the sample primarily consisted of CBT-licensed psychotherapists (303/350, 86.6%), indicating that other psychotherapeutic orientations, such as psychoanalysis (9/350, 2.6%), were underrepresented. This observation may be attributed to the unrestricted sampling procedure, which likely attracted more participants from specific psychotherapeutic orientations who were generally interested in IMIs. Second, the majority of the sample had no prior experience with IMIs (221/350, 63.1%). This indicates that many responses reflect concerns or potential advantages of IMIs that may not be grounded in actual experiences from mental health care practices. Future studies should include a more heterogeneous mix of psychotherapeutic orientations or focus solely on psychotherapists with experience using IMIs to allow for a comparison of the present findings. The results presented reflect opinions gathered in a bottom-up manner and analyzed in an exploratory fashion. Therefore, it does not claim to represent any scientifically proven relationships between entities. This study was conducted in Germany, where IMIs have recently been introduced to the health care system as digital health apps available by prescription [43]. Consequently, the results of this study apply only to the German context and cannot be generalized. Additionally, the responses were obtained anonymously through an online survey, which limited the researchers’ ability to clarify the meaning of the questions or pursue further inquiries. In-depth expert interviews with psychotherapists who have extensive experience and knowledge in utilizing IMIs could yield valuable insights in the future. Such studies could reveal how to develop future IMIs and understand their associated working mechanisms in both clinical practice and research. As perceived risks for patients may vary depending on the stakeholder’s perspective, consulting patients’ opinions to gather critical information on addressing actual risks is essential.

### Conclusions

This study offers comprehensive insights into the attitudes of psychotherapists toward IMIs. This area of psychotherapy has been less explored, particularly regarding the efficacy of specific IMIs in reducing psychiatric symptoms. The acceptability of IMIs, not only among patients but also among psychotherapists, can be successfully achieved by carefully considering the opinions of all stakeholders involved in psychotherapeutic treatment. Regarding the 4 overarching categories—Treatment Resources, Technology, Applicability of IMIs, and Perceived Risks and Barriers—several significant implications emerged: Many psychotherapists welcomed IMIs as a new tool to support conventional psychotherapy (eg, blended therapy) in treating mental disorders; however, they expressed skepticism about using IMIs as a substitute for traditional psychotherapy. Currently, unresolved technological issues and perceived shortcomings in data protection hinder the use of IMIs. As several factors (eg, the rationale for therapy or the applicability to specific disorders) may influence the efficacy of IMIs, it is crucial to promote future research on the boundary conditions of IMI use (eg, to develop robust contraindications and indications). Additionally, health insurance companies and other stakeholders should explore ways to reimburse training programs and supervision to alleviate the costs and time spent on research for psychotherapists. It would also be beneficial to find more effective methods for making recent research findings readily available to practicing psychotherapists and integrating them into the development of new IMIs.

The increasing use of IMIs in recent years has brought several benefits, opportunities, and facilitating conditions, but also presents risks, as indicated by this study on practicing psychotherapists. While IMIs have become an integral part of today’s psychotherapeutic health care system, it is crucial to maintain an ongoing dialogue among insurance companies, developers, therapists, and patients to address emerging needs and concerns and ultimately enhance the effectiveness of IMIs. However, it must be clearly stated that IMIs are only a support for the therapeutic process and not a panacea for the increasing cases of mental illnesses or the shortage of licensed therapists. Further health policy decisions are certainly needed beyond the scope of this study and the use of IMIs. Therefore, this study should be understood as a contribution to psychotherapeutic care and is just one of many steps toward sustainably improving treatment options for affected individuals.

## Appendix

Multimedia Appendix 1 Questions administered and code segments.

Multimedia Appendix 2 Number of prescriptions by disorder.

## Abbreviations

CBT: cognitive behavioral therapy
IMI: internet- and mobile-based intervention

## Data Availability Statement

The data supporting these findings are available on request from the corresponding author.
