# Supplementary material for: Exploring Psychotherapists’ Attitudes on Internet- and Mobile-Based Interventions in Germany: Thematic Analysis
Source: JMIR Form Res. 2024 Nov 7;8:e51832. doi: 10.2196/51832 (PMC11582492; doi:10.2196/51832)
Supplement: Multimedia Appendix 1 [file formative_v8i1e51832_app1.docx]

**Supplementary Material**

**Table 1**

*Full list of administered questions regarding therapists attitudes towards internet- and mobile-based interventions and prior usage of IMIs.*

| Number | Question Type | Question |
| --- | --- | --- |
| 1 | Open | “Does your working environment support the use of new technologies and if so, how?“ |
| 2 | Open | "To what extent does your work environment not show support for the use of new technologies?" |
| 3 | Open | "What is missing so that your work environment would support the use of new technologies?" |
| 4 | Open | "What aspects of working with digital health apps have you found disruptive so far?" |
| 5 | Open | “Was there something that kept you from using IMIs in your psychotherapeutic practice and if so, what?” |
| 6 | Open | “What challenges could the usage of IMIs encounter?” |
| 7 | Open | “What risks could the usage of IMIs encounter?” |
| 8 | Open | “Which requirements need to be met for you to use IMIs in your psychotherapeutic practice?” |
| 9 | Open | “Which functions do IMIs need to include for you to be useful in psychotherapy?” |
| 10 | Open | “Are there psychotherapeutic orientations for which IMIs are (particularly/rather not) suited and if so, which?" |
| 11 | Open | "For which interventions do you think the use of digital health apps has the greatest potential?" |
| 12 | Open | "For which groups of disorders do you think Digital Health apps are not appropriate and why?" |
| 13 | Open | "If you want to describe specific experiences, you can briefly state what they were here." |
| 14 | Open | "For which areas would you find the use of digital health apps most useful?" |
| 15 | Closed | "Have you prescribed IMIs yet?" |
| 16 | Closed | "For which disorders have you already prescribed Digital Health apps?" |
| 17 | Closed | "In your estimation, how many patients drop out or don't use IMIs after they are prescribed?" |

*Note.* IMI: internet- and mobile-based interventions.

**Table 2**

*Code segments falling into the category “Applicability of IMIs” and the corresponding core themes “Disorder-related Limitations”, “Facilitating Structures”, “Psychotherapeutic Specialization”, and “Role in Mental Health Care”.*

| Core theme | Sub-theme | n |
| --- | --- | --- |
| Disorder-related Limitations |  | 444 |
|  | Personality disorders | 75 |
|  | Psychotic disorders | 62 |
|  | Post-traumatic stress disorder | 38 |
|  | Depression | 31 |
|  | Dependent of the respective patient | 27 |
|  | Acute crisis (e.g., suicidality) | 24 |
|  | Disorders regarding relationships | 22 |
|  | Cognitive impairment | 21 |
|  | No suitable disorder | 20 |
|  | Severe symptoms | 19 |
|  | Anxiety | 19 |
|  | Missing motivation/awareness of problem | 14 |
|  | Dissociation | 13 |
|  | High complexity, comorbidity | 12 |
|  | Age-dependent | 12 |
|  | Addiction | 11 |
|  | Bipolar disorders | 9 |
|  | Obsessive-compulsive disorder | 6 |
|  | Eating disorders | 5 |
|  | Others | 4 |
| Facilitating Structures |  | 330 |
|  | Support by employers | 172 |
|  | Collegial exchange and openness | 60 |
|  | Digital diagnostics and monitoring | 25 |
|  | Support by health insurance companies | 20 |
|  | Training | 18 |
|  | Usage of IMIs | 15 |
|  | Incorporation in research | 8 |
|  | Guidelines | 6 |
|  | Technological support | 6 |
| Psychotherapeutic Specialization |  | 234 |
|  | Not suitable for psychoanalysis | 89 |
|  | Not suitable for psychodynamic | 69 |
|  | Not suitable for systemic therapy | 26 |
|  | Not suitable for CBT | 15 |
| Role in Mental Health Care |  | 112 |
|  | No substitute for therapy | 50 |
|  | As an add on | 40 |
|  | Reduced worth/rationalization | 14 |
|  | Bridging waiting times | 5 |
|  | Others | 3 |

*Note.* n = Number of segments allocated to a code. CBT = Cognitive Behavioral Therapy.

**Table 3**

*Code segments falling into the category “Treatment Resources” and the corresponding core themes “(Lack of) Information” and “Costs and Effort”.*

| Core theme | Sub-theme | n |
| --- | --- | --- |
| (Lack of) Information |  | 451 |
|  | Provided information | 157 |
|  | Knowledge of psychotherapists | 86 |
|  | Evidence and guidelines | 71 |
|  | Testing facilities | 66 |
|  | Training | 29 |
|  | Oversupply | 14 |
|  | Experience | 10 |
|  | Advertising | 6 |
|  | Informing patients | 5 |
|  | Support from companies | 4 |
|  | Guidelines | 3 |
| Costs and Effort |  | 194 |
|  | (Initial) training | 54 |
|  | General effort | 52 |
|  | Economic interests | 32 |
|  | Prescribing and accounting | 29 |
|  | High cost | 24 |
|  | Missing support by health insurance | 3 |

*Note.* n = Number of segments allocated to a code.

**Table 4**

*Code segments falling into the category “Technology” and the corresponding core themes “Technical Constraints”, “Technical Requirements and Functions”, and “Data Protection and Privacy”*

| Core theme | Sub-theme | n |
| --- | --- | --- |
| Technical Constraints |  | 167 |
|  | Usability | 69 |
|  | Customizability | 54 |
|  | Technical problems | 26 |
|  | Increased screen time/addiction risk | 10 |
|  | Digital infrastructure | 8 |
| Technical Requirements and Functions |  | 157 |
|  | Interaction with psychotherapist | 54 |
|  | Evaluation and feedback | 19 |
|  | Diary | 19 |
|  | Video and sound recording | 14 |
|  | Reminders and calendar | 12 |
|  | Symptom tracking | 12 |
|  | Therapeutic elements (e.g., psychoeducation, exposition) | 10 |
|  | Emergency function | 9 |
|  | Others | 8 |
| Data Protection and Privacy |  | 115 |
|  | Privacy | 108 |
|  | Transparency | 7 |

*Note.* n = Number of segments allocated to a code.

**Table 5**

*Code segments falling into the category “Perceived Risks and Barriers”, divided into the two core themes “Perceived Risks and Barriers for Psychotherapists” and “Perceived Risks and Barriers for Patients”.*

| Core theme | Sub-theme | n |
| --- | --- | --- |
| For Psychotherapists |  | 216 |
|  | Acceptance and motivation | 110 |
|  | Integration into the daily routine | 51 |
|  | Control | 19 |
|  | Contact with patient | 18 |
|  | Regression and legal concerns | 14 |
|  | Technical competencies | 4 |
| For Patients |  | 114 |
|  | Individualization and fit | 51 |
|  | Barriers | 32 |
|  | Acceptance/compliance | 21 |
|  | Understanding | 14 |
|  | Overload | 14 |
|  | Relationship | 8 |
|  | Others | 4 |

*Note.* n = Number of segments allocated to a code.

**Figure 1**

*Translation of the description of internet- and mobile-based interventions, given to psychotherapists who participated in the study, before participation.*

Since the end of 2020, doctors and psychotherapists have been able to prescribe so-called digital health applications (DiGA) to their patients. These certified medical devices use digital technologies (e.g. apps) for the detection, monitoring, treatment, alleviation, or compensation of health complaints (Section 33a (1) SGB V). They can be used by patients independently or together with doctors or psychotherapists. DiGAs must demonstrate a positive effect on care (medical benefit or patient-relevant structural/procedural improvement) and be registered with the Federal Institute for Drugs and Medical Devices. They can also be registered on a trial basis for 12-24 months, during which time proof of efficacy must be provided. Their costs are covered by the health insurance companies.
