# Supplementary figures and images for: Exploring Psychotherapists’ Attitudes on Internet- and Mobile-Based Interventions in Germany: Thematic Analysis
Source: JMIR Form Res. 2024 Nov 7;8:e51832. doi: 10.2196/51832 (PMC11582492; doi:10.2196/51832)

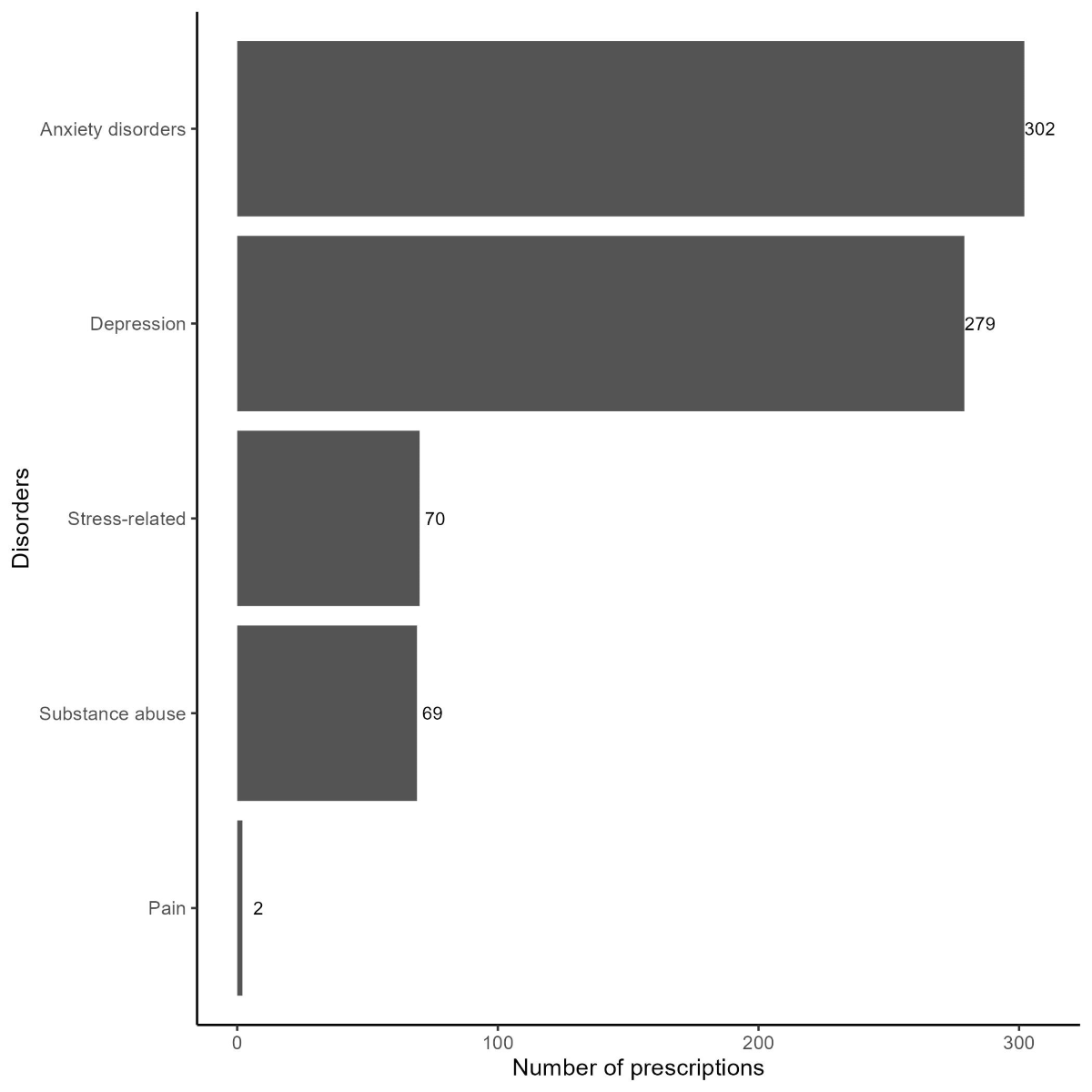

Supplement: Multimedia Appendix 2 [file formative_v8i1e51832_app2.png]
